# Research priorities for shoulder surgery: results of the 2015 James Lind Alliance patient and clinician priority setting partnership

**DOI:** 10.1136/bmjopen-2015-010412

**Published:** 2016-04-11

**Authors:** Amar Rangan, Sheela Upadhaya, Sandra Regan, Francine Toye, Jonathan L Rees

**Affiliations:** 1 The James Cook University Hospital, Middlesbrough, UK; 2 James Lind Alliance Advisor, NIHR, London, UK; 3 James Lind Alliance Project Manager and Hub Co-ordinator, NIHR Oxford Biomedical Research Centre, Oxford, UK; 4 Nuffield Orthopaedic Centre, Oxford University NHS Trust, Oxford, UK; 5 Nuffield Department of Orthopaedics, Rheumatology and Musculoskeletal Science, NIHR Oxford Biomedical Research Unit, University of Oxford, Oxford, UK

**Keywords:** James Lind Alliance, uncertainties, SURGERY

## Abstract

**Objective:**

To run a UK based James Lind Alliance Priority Setting Partnership for ‘Surgery for Common Shoulder Problems’.

**Setting:**

This was a nationally funded and conducted process. It was organised from a musculoskeletal research centre and Biomedical Research Unit in Oxford.

**Participants:**

UK shoulder patients, carers and clinicians, involved in treating patients with shoulder pain and shoulder problems that might require surgery.

**Interventions:**

These were national electronic and paper surveys capturing treatment uncertainties that are important to shoulder patients, carers and clinicians.

**Outcome measures:**

The outcomes relevant to this study were the survey results and rankings.

**Results:**

The process took 18 months to complete, with 371 participants contributing 404 in scope questions. The James Lind process then produced a final 10 research priorities and uncertainties that relate to the scope of ‘Surgery for Common Shoulder Problems’.

**Conclusions:**

The final top 10 UK research priorities have been produced and are now being disseminated to partner organisations and funders to guide funding of shoulder research for the next 5–10 years on topics that are important to patients, their carers and clinicians.

**Strengths and limitations of this study**

- The study adheres to the structured process and principals of the James Lind Alliance.
- The process and study are patient-centric.
- The process and study have produced the top 10 research treatment uncertainties in relation to surgery for common shoulder problems.
- While the process and study recommend those research priorities that are important, there is no guarantee of research funding.
- This is the first nationally-funded priority setting partnership in orthopaedics and this funding model is now being adopted by other orthopaedic specialty societies.

## Introduction and background

The James Lind Alliance (JLA) is now hosted by the UK National Institute for Health Research (NIHR) Evaluation, Trials and Studies Coordinating Centre (NETSCC). Its aim is to provide an approved process^1^ that enables patients and clinicians to work together to agree on the most important treatment uncertainties in a particular field of interest. It then publishes and disseminates these priority areas to partners and funding organisations in order to influence the prioritisation of future research.

Shoulder pain is the third most common musculoskeletal symptom suffered by patients in primary care with 2.4% adult prevalence for general practitioner (GP) consultations each year in the UK.^2–4^ As such, referrals to secondary care are increasing and, with employment implications, cost estimates of £100 million have been suggested. Some shoulder operations have increased 700% in 8 years.^5^ With most aspects of health provision, there remains a lack of high-level evidence for management pathways and therefore uncertainty still exists about some aspects of shoulder surgery, such as when the best time to operate on patients with shoulder problems is, which patients need surgery and which patients are best treated non-operatively.

In 2013, funding was raised to initiate and run a JLA Priority Setting Partnership (PSP) for ‘Surgery for Common Shoulder Problems’. This PSP was set up as a national model for orthopaedics, with funding provided by the relevant national professional organisations, namely the British Elbow and Shoulder Society (BESS) and the British Orthopaedic Association. Further financial support was provided by NIHR through the NIHR Oxford Musculoskeletal Biomedical Research Unit and the NIHR Oxford Biomedical Research Centre. It was hoped that this initiative would also encourage other orthopaedic societies to follow a similar funding model in order to help shape the relevance of future orthopaedic and musculoskeletal research in the UK, by engaging with and involving patients, carers and other health professionals involved in the care of these patients. This surgical shoulder PSP and its model of funding by the national professional organisations was fully supported by NETSCC, and the PSP application was approved in November 2013.

The aim of the ‘Surgery for Common Shoulder Problems’ PSP was to identify the unanswered questions about surgical treatments for common shoulder problems by:

- Working with patients, clinicians and allied health professionals to identify treatment uncertainties about different types of shoulder surgery including when to operate, and which patients are best treated with surgery.
- Agreeing by consensus on a prioritised top 10 list of uncertainties.
- Publicising the results of the PSP and process, and taking these results to research commissioning bodies.

## Method and stages

PSPs follow a structured process that needs to be adhered to in order to obtain final approval of the results and endorsement of the top 10 research priority areas by the JLA.^1^ First, a JLA adviser (SU) was appointed by NETSCC to the PSP to work with the clinical and specialist lead (JR) to set up the PSP Steering Group. This group provided oversight and management of the PSP. The Steering Group was made up of the most relevant stakeholders and included patients; physiotherapists; GPs; shoulder surgeons; anaesthetists and pain control experts; orthopaedic nurses and an academic clinician (AR). Finally a JLA co-ordinator and a data analyst also joined the group. With the Steering Group in place, the following JLA PSP stages took place between January 2014 and July 2015. Meetings were centralised in Oxford for practical resource reasons with some Steering Group meetings also taking place via conference calls.

### Identification and invitation of potential partners

Potential partner organisations were identified, contacted and informed of the establishment and aims of the ‘Surgery for Common Shoulder Problems’ PSP. Invited organisations and individuals represented people who had undergone hospital treatments for common shoulder problems, carers of people who had had hospital treatments for common shoulder problems, medical doctors, nurses and allied health professionals with clinical experience of treating patients with common shoulder problems, and GPs with clinical experience of referring patients with common shoulder problems for hospital care. These groups were invited to attend and participate in the initial stakeholder meeting, to be partners and to help disseminate surveys and results.

### Initial stakeholder meeting/awareness raising

The initial stakeholder meeting had several key objectives: to welcome and introduce potential members of the PSP; to present the proposed plan for the PSP; to initiate discussion, answer questions and address concerns; to identify those potential partner organisations that would commit to the PSP and identify the contact representatives; to establish principles on which an open, inclusive and transparent mechanism could be based for contributing to, reporting, and recording the work and progress of the PSP.

### Identifying treatment uncertainties

For common shoulder problems, each partner identified the method for soliciting from its members, questions and uncertainties of practical clinical importance relating to different types of shoulder surgery including which patients might be best treated with or without surgery.

### Refining questions and uncertainties

The Steering Group allocated responsibility for this stage and two members (JR and FT) ran the data management and analysis, while the Steering Group and JLA provided guidance, to ensure accountability and transparency. The consultation process produced ‘raw’ unanswered questions. These raw questions were assembled and categorised by the data analysts into ‘collated indicative questions’, which were made clear and understandable to all. Similar or duplicate questions were combined. Uncertainties, not adequately addressed by previous research, were recorded and prepared for entry into a ‘Surgery for Common Shoulder Problems’ section within the UK Database of Uncertainties about the Effects of Treatments (UK DUETs—http://www.library.nhs.uk/duets). This ensured that the uncertainties were retained even if they did not make the top 10.

### Prioritisation—interim and final stages

The aim of the final stage of the PSP was to prioritise through consensus the identified uncertainties. This was carried out by the Steering Group and the wider partnership represented by patients and clinicians. For the interim stage, a long list of uncertainties was reduced to a shorter list by means of an online survey and Steering Group meeting. This online survey was written in common language and adopted the principals of a red light, amber light and green light system, with the responses allowed being ‘yes’ (important), ‘no’ (not important) and ‘unsure’. This method of interim prioritisation has been used by other PSP's and allows the Steering Group to assess all the responses from all stakeholder groups. This fully informs the interim prioritisation with ‘green light’ responses to the same questions from different stakeholder groups, indicating a high-level of importance of that uncertainty.

The final prioritisation stage to reach the 10 prioritised uncertainties, was conducted by a face-to-face meeting, using group discussions and plenary sessions. All 25 uncertainties were discussed, considered and ranked by break-out groups with equal representation of stakeholders. Each group was led by an independent JLA advisor and the groups were rotated throughout the day with the process continuing until there was agreement over the top 10 uncertainties. The JLA facilitated the entire final day ensuring the JLA process was followed and ensuring transparency, accountability and fairness. All participants needed to declare their interests in advance of this final meeting.

## Results

The initial national survey produced 652 questions from 371 patients, carers and clinicians. When each question was reviewed, 404 fell within the predefined scope of this PSP. There were a number of duplications highlighting the importance in some areas and allowing the combining of these duplications to produce 143 questions.

**Figure 1:**
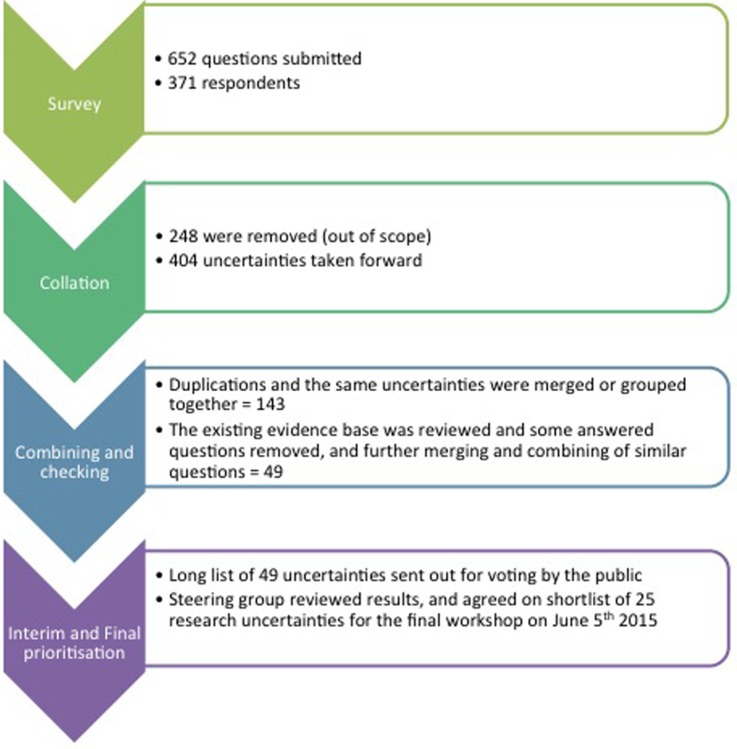
Flow chart of priority setting partnership process indicating the number of questions at each stage.

With further merging of questions that were essentially asking the same question and by taking into account which questions were asked by different demographic sources and then ensuring any remaining questions were true uncertainties, 49 questions were finally produced. These 49 questions then went out for the interim prioritisation by the electronic web-based survey in March 2015. This interim prioritisation produced a shortlist of 25 uncertainties that underwent final prioritisation at a workshop in Oxford on 5 June 2015. Figure 1 highlights the stages and processing of the questions.

Final prioritisation resulted in the top 10 uncertainties for surgery for common shoulder problems. While a view was taken at the final prioritisation meeting that these 10 priorities are equally important and would be presented as bulleted priorities, the order in which they appear in box 1 represents their ranked positions and scores on the final day. Ranking is important to some funders who prefer to assess the ranking order when considering funding research questions extracted from these priority areas.

Box 1 **Top 10 research priority areas from the James Lind Alliance Priority Setting Partnership for surgery for common shoulder problems**

*Top 10 questions*

- For the main shoulder conditions of arthritis, frozen shoulder, impingement, rotator cuff tears and instability, can you predict which patients will do well with surgery to help them decide on whether to have surgery or not?
- In patients with 3 and 4 part proximal humeral fractures, what is the long-term outcome of reverse total shoulder replacement compared to hemiarthroplasty?
- Does arthroscopic subacromial decompression surgery in patients with degenerative rotator cuff tendon problems improve outcome and prevent further tendon degeneration and tears compared to patients with no surgical intervention?
- Does early mobilisation and physiotherapy after shoulder surgery improve patient outcome compared to standard immobilisation and physiotherapy?
- In patients with shoulder arthritis is a hemiarthroplasty or a total shoulder replacement or a reverse replacement most effective?
- Are patients (including older age groups) with rotator cuff tendon tears in their shoulder best treated with surgery or physiotherapy?
- How can we ensure that patients see the right doctors and clinicians promptly and correctly, and does this lead to better outcomes?
- In patients with frozen shoulder, does early surgery improve outcome compared to non-surgery treatments such as injection and dilatation?
- In patients with newly diagnosed calcific tendinitis (calcium in a shoulder tendon), is early surgical intervention more clinically effective than non-operative treatments?
- Do patients with partial thickness rotator cuff tendon tears benefit more from a surgical repair compared to a decompression and debridement alone?

## Discussion

This JLA PSP was funded and set up in response to what is currently a pendulum swing for research funding bodies towards prioritising research questions by engaging patients in the selection of priority areas. This is to ensure that priority areas chosen are important to patients, carers and clinicians, and not only to researchers and academics. The JLA is the process and method that has been approved in the UK for such priority setting partnerships.

At present, a number of very diverse PSPs have been completed with many more underway. The processes are the same but there are different challenges and variations to the methods needed within these processes for differing PSPs. Our observations are that duration and costs of running a PSP can vary from one partnership to another. This can depend on a number of variables, but breadth of topic is critical in depicting duration of the process as well as for finding out whether the priority outcomes will be useful and are likely to be funded. This is a balance that needs careful consideration. We would recommend to anyone wishing to run a JLA PSP to consider the topic very carefully, as duration affects cost, but selecting a narrow topic is not necessarily cost-effective. We found the breadth of this shoulder surgery PSP to be probably at the limit of what is practical. Delivering it in an 18-month window has required a large amount of resource and professional time. While we received >600 questions that required processing, some PSPs receive well over 1000 questions, which would clearly impact resource requirements and duration, and highlights the reasons for variability seen in different PSPs. The final cost of this PSP was £25 000.00. While this serves as a guide, it should be noted that costs were kept down by holding meetings within the senior authors’ institution and by keeping data processing in-house. For those considering a PSP, we would recommend our guideline figure as an absolute bare minimum, and all factors mentioned above should be carefully considered and taken into account.

An important aspect of a JLA PSP is the transparent process and, as such, all data are maintained in a manner that can be tracked back at any point to the original questions and demographic source. The power and usefulness of running a PSP and producing the top 10 priority areas have been highlighted by others who have had all 10 of their research priorities funded. These facts make for compelling reasons to run a PSP, and involving the relevant stakeholders in deciding on what research should be funded would seem to be an effective and sustainable model. It is only likely to be overridden by research topics into treatments that have a profound national health cost implication. Overall, we found this JLA PSP a positive and worthwhile experience, and our patient representatives, in particular, found it thoroughly rewarding. The results of the shoulder surgery PSP were announced and presented by one of the authors (JR) on Thursday 25 June 2015, at the BESS annual conference. They are now being disseminated via formal publication and social media. The findings of the ‘Surgery for Common Shoulder Problems' PSP will be reported to funding and research agenda setting organisations such as the NIHR Evaluation, Trials and Studies Coordinating Centre (NETSCC), which includes the NIHR Health Technology Assessment Programme, and the Medical Research Council, as well as the major research funding charities.
